# Supplementary material for: Spatiotemporally resolved multivariate pattern analysis for M/EEG
Source: Hum Brain Mapp. 2022 Mar 18;43(10):3062–85. doi: 10.1002/hbm.25835 (PMC9188977; doi:10.1002/hbm.25835)
Supplement: Supplementary file 1 — Appendix S1 Supporting Information. [file HBM-43-3062-s001.pdf]

Supplementary information:

Supplementary Text:

1. Model verification using simulated data
2. Test-set state timecourse fitting
3. Model sensitivity to number of states
4. Comparison with inverted decoding model predictions

Supplementary Figures:

1. Ground truth simulations and inferred STRM-Classification model parameters
2. Ground truth simulations and inferred STRM-Regression model parameters
3. The full cross validation procedure
4. Comparing accuracy of different cross validated state fitting methods
5. Robustness of STRM-Classification over different parameter values
6. Robustness of STRM-Regression over different parameter values
7. Comparing forward model parameters computed directly or using post-hoc methods applied to linear classifiers

1. Model verification using simulated data

To verify that the model inference procedure functioned as intended, we simulated data from the generative model such that real 'ground truth' parameter values were known; inferred the posterior distribution for these parameters; and verified the accuracy. These are shown below; for further details and other simulations see (Higgins, 2019).

Case 1: STRM-Classification

First we simulated data in two dimensions under two categorical stimuli (along with an intercept term) switching between two sequential latent states ($P = Q = K = 2$). We simulated trials of length T=50 timesteps, with all trials beginning in state 1 and then transitioning to state 2 at some randomly generated timepoint within the trial; transition times were sampled from a uniform distribution on the interval $[2,49]$. We simulated a total of 10 trials, with 5 of each stimulus. Stimulus activation patterns W_k were randomly generated from a standard normal distribution; state covariance patterns Σ_k were generated from a Wishart distribution with identity matrix mean and 2 degrees of freedom. Figure S1A plots the data, with red and green colors denoting the class active for that sample. On the right hand side is the ground truth latent state activation simulated for each trial.

Figure S1B shows the inferred state timecourses alongside their true values; these closely match, demonstrating that the model has recovered almost exactly the ground truth state timecourses used to generate the data. Figure S1C plots the data assigned to each latent state, highlighting that these are more clearly separable than the data in figure S1A. Figure S1D shows the mode of the posterior distributions inferred for the model, with cross markers denoting the posterior mean of W and contour plots showing the posterior mode of the covariance matrix Σ. When compared to the corresponding ground truth model values alongside each plot, they can be seen to match the data and the generative parameter values closely.

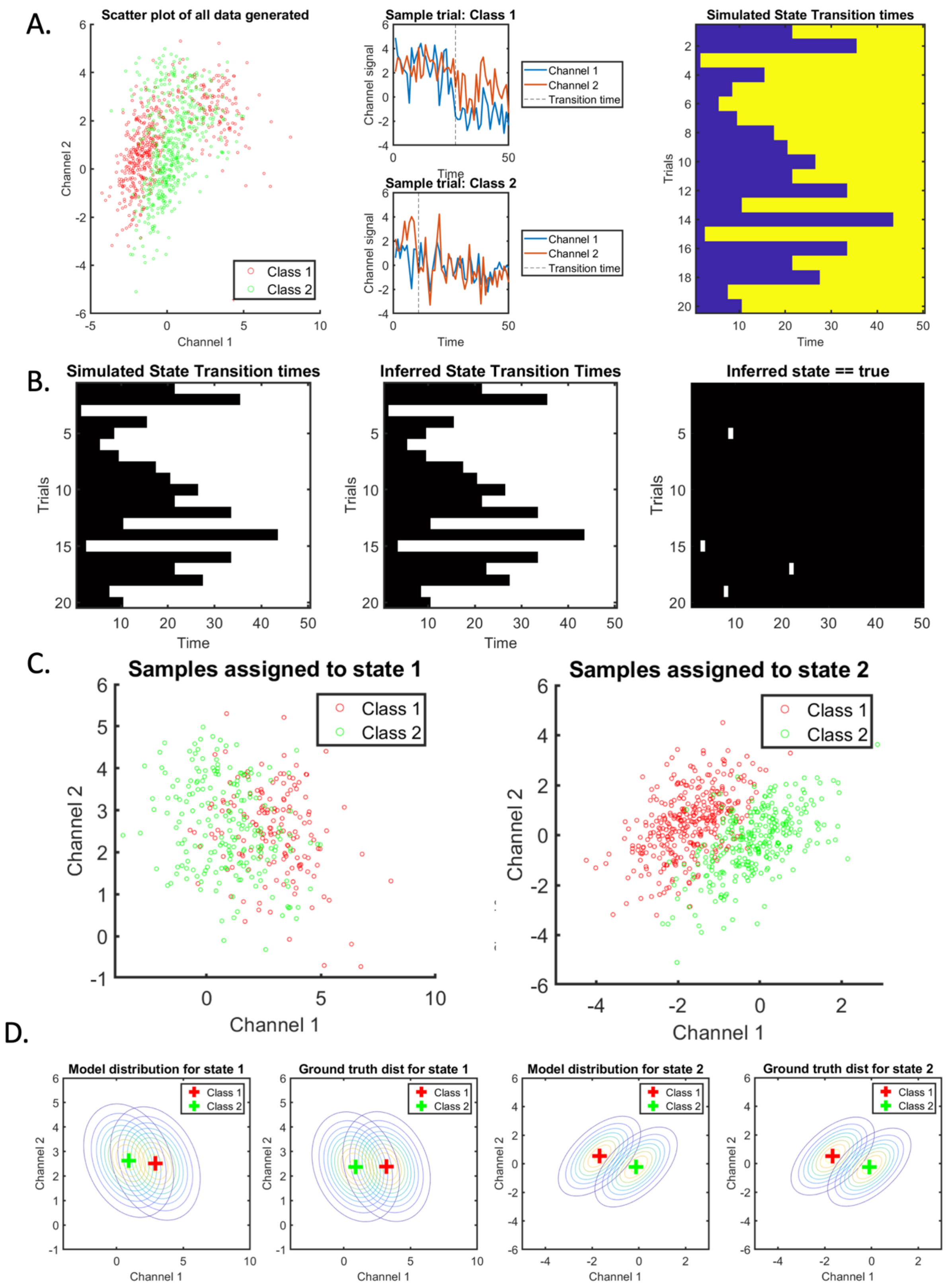

Figure S1: Ground truth simulations and inferred STRM-Classification model parameters. *A. The generated data. We randomly sampled parameters from the generative model as outlined in the text; the scatter plot shows the distribution of all datapoints (collapsing over all trials and timepoints), with green and red dots denoting the class the data pertains to. To highlight the temporal evolution, channel data from two sample trials are also plotted against time (one from each class), with the transition time between the two latent*

states highlighted. B. Inferred state timecourses match simulated ground truth: Plot on the left shows the simulated state timecourses as a raster plot (each row is a trial, each column is a timepoint, and the colouring of white/black denotes that state 1 or 2 respectively is active); the middle plot shows the inferred latent state timecourses, which qualitatively match the ground truth; right plot confirms the inferred latent state matches the ground truth for all but 4 samples (in which the inferred state switch time was out by one timepoint). C. The model separates datapoints into latent states that maximise model fit. Plots show the datapoints assigned into each of latent states 1 and 2 respectively; compared to the scatter plot in A, these are more clearly separable by class. D. Inferred model parameters match ground truth values; plots show the model distribution parameters – i.e. the inferred class mean and covariance – alongside the ground truth parameter values, demonstrating a tight fit.

Case 2: STRM-Regression

We then simulated from the STRM-Regression model. We again simulated data in two dimensions, with a single continuous valued regressor (along with an intercept term). Regressor values generated for each trial were drawn from a standard normal distribution (the regressor value was fixed for the duration of the trial). We again simulated trials of length 50 samples, with all trials beginning in state 1 and then transitioning to state 2 at some randomly generated timepoint within the trial; transition times were sampled from a uniform distribution on the interval $[2,49]$. We simulated a total of 20 trials. Stimulus activation patterns W_k were randomly generated from a standard normal distribution; state covariance patterns Σ_k were generated from a Wishart distribution with identity matrix mean and 2 degrees of freedom. Figure S2A plots the data, with coloured shading indicating the value of the regressor as indicated on the colour bar.

Figure S2B shows the inferred state timecourses alongside their true values; these closely match, demonstrating that the model has recovered almost exactly the ground truth state timecourses used to generate the data. Figure S2C plots the data assigned to each latent state, in each of which a clear and distinct regressor encoding direction and noise pattern is visible. Figure S2D shows the mode of the posterior distributions inferred for the model, with vector arrows denoting the direction and scaling of the regressor encoding direction W and contour plots showing the posterior mode of the covariance matrix Σ. When compared to the corresponding ground truth model values alongside each plot, they can be seen to match the data and the generative parameter values closely.

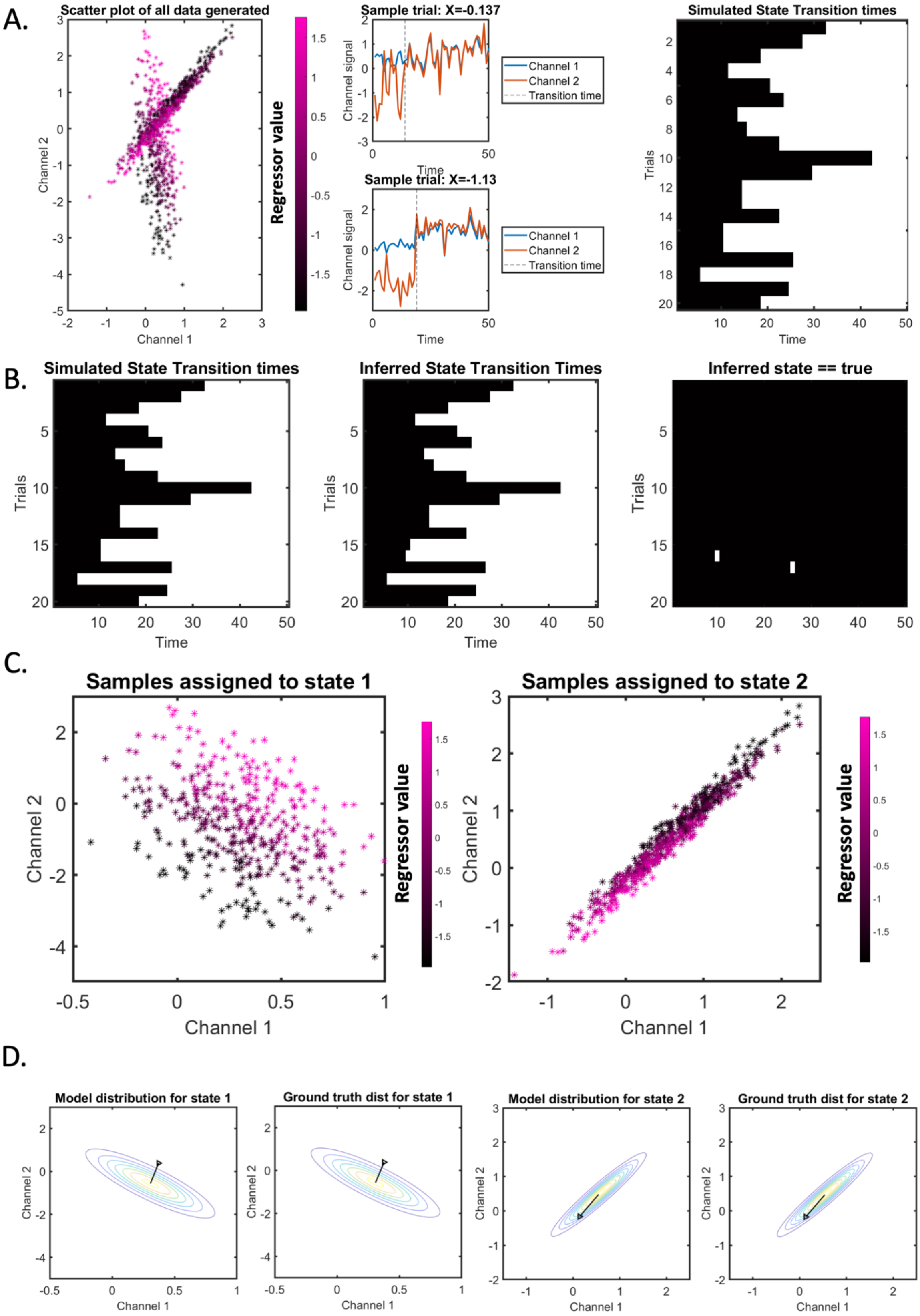

Figure S2: Ground truth simulations and inferred STRM-Regression model parameters. A. *The generated data. We randomly sampled parameters from the generative model as outlined in the text; the scatter plot*

shows the distribution of all datapoints (collapsing over all trials and timepoints), with colour denoting the value of the sole regressor X associated with each datapoint. To highlight the temporal evolution, channel data from two sample trials are also plotted against time (with the regressor value indicated for each), with the transition time between the two latent states highlighted. Right: ground truth simulated state timecourses. B. Inferred state timecourses match simulated ground truth: Plot on the left shows the simulated state timecourses as a raster plot (each row is a trial, each column is a timepoint, and the colouring of white/black denotes that state 1 or 2 respectively is active); the middle plot shows the inferred latent state timecourses, which qualitatively match the ground truth; right plot confirms the inferred latent state matches the ground truth for all but 2 samples (in which the inferred state switch time was out by one timepoint). C. The model separates datapoints into latent states that maximise model fit. Plots show the datapoints assigned into each of latent states 1 and 2 respectively; compared to the scatter plot in A, the distinct linear relationships with the regressor can be more clearly identified. D. Inferred model parameters match ground truth values; plots show the model distribution parameters – i.e. the inferred data covariance and a vector indicating the direction of regressor encoding W – alongside the ground truth parameter values, demonstrating a tight fit.

2. Test-set state time course fitting

When using cross validation to gauge the model's performance on held-out data (i.e. for the sections computing model predictive accuracy metrics plotted in Figure 6 and Figure 9 of the main text), we face the problem that we cannot know a-priori the correct values of the state timecourses for the held out test set; the STRM model differs from standard MVPA approaches in that these model parameters are trial-specific.

Whilst we are unable to infer these parameters directly using the model, we can resort to procedures that estimate them. As outlined in the text we used a linear regression model to estimate the state timecourses from the data directly. We explain this method in more detail here and justify this choice over several alternatives.

Specifically, as in Figure S3, the cross validation procedure involves (i) partitioning the data into training and test folds; (ii) learning all STRM model parameters from the training set; (iii) computing the expected value of the observation model (ie non-trial-varying) parameters $\widetilde{W}_{1:K}$ and $\tilde{\Sigma}_{1:K}$ from the posterior; (iv) estimating a linear relationship between the training data latent state parameters (ie the parameters that are unique to each trial: $\tilde{z}_t$ for t in the training set); note we train a different model for each timepoint within the trial (v) using this linear model to estimate the test set latent state parameters (ie $\tilde{z}_t$ for t in the test set); (vi) computing the predictions of the model for the test set and computing the accuracy of these, and (vii) repeating for each cross validation fold.

We did investigate several different methods for estimating the latent state parameters $\tilde{z}_t$ from the data, which we briefly justify here: overall, we found that the regression model was robust over different data types, and fast (an important consideration given the number of folds over which these computations had to be repeated). In figure S4, we compare the following methods:

(i) The regression method outlined in the main text and above;

(ii) Computing the *mean state timecourse* $\tilde{z}_t$ observed over trials in the training set as the each test set trial's state timecourse. Note that in this case there are no inter-trial differences in the encoding model fit as the mean over trials does not vary.

(iii) Learning an *equivalent unsupervised model*. This procedure involves holding the inferred state timecourses for the training data fixed and learning the parameters of an unsupervised model of the type used in (Vidaurre et al., 2016); specifically, inferring a single mean and covariance across all channels of the data for each state, with no knowledge of the design matrix. Such a model could then be fit to the test set data to infer state timecourses in an unbiased way.

Alternatively, a principled method that may appear more suited to our general Bayesian approach consists of inferring the joint distribution over latent state timecourses *and* design matrix entries, and then marginalising out over the latent state distribution at each timepoint (as outlined in detail in section 3.4.2 of Beal, (2003)).

Unfortunately, this principled approach would (i) only be tractable for the case of the STRM-classification model, and (ii) would only be computationally feasible after imposing an additional assumption for the purposes of the joint probability that the stimulus never varied over the course of the trial. Whilst this assumption is true in practice for our data, it could be considered unfair as a comparison to timepoint-by-timepoint decoding methods, which by definition provide predictions independently at each point in time.

As an example, Figure S4 shows the accuracy achieved fitting a STRM model with K=12 states, using each of these methods to estimate the held-out test set state timecourse $\tilde{z}_t$. Across the three different estimation methods evaluated, using the mean state timecourse achieves slightly better results than using the regression model; whilst using the equivalent unsupervised model achieves significantly poorer performance.

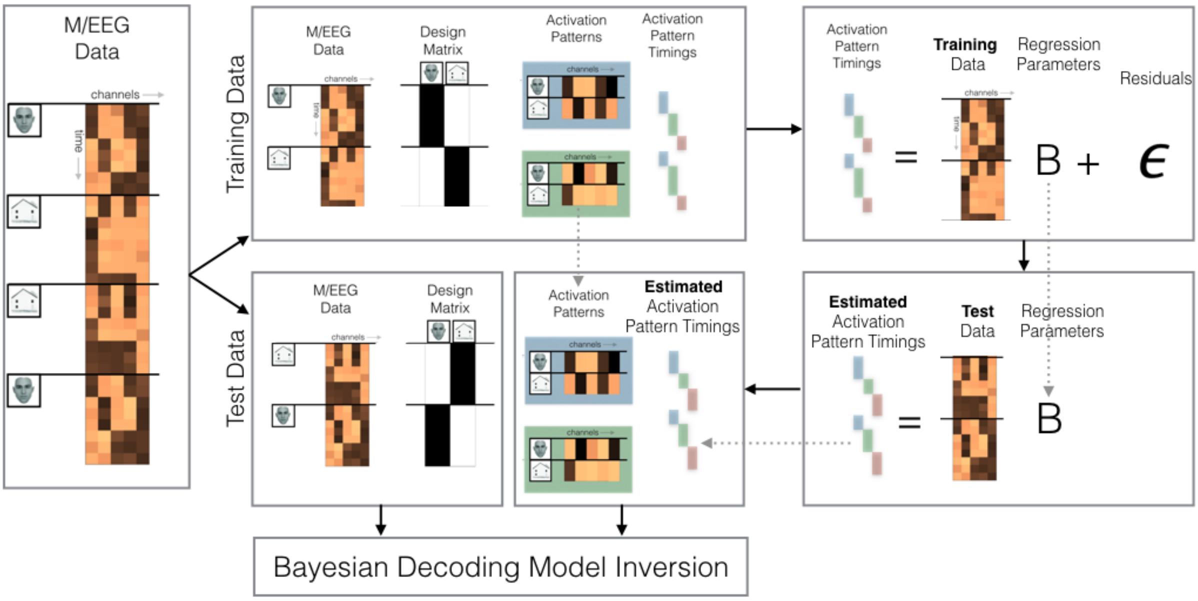

Figure S3: The full cross validation procedure. *Cross validation involves partitioning the data into training folds and test folds (left hand side), and training the STRM model on the training data. The STRM model includes some parameters that can be used directly on the test set (the activation patterns) and some parameters that are unique to each trial (the activation pattern timings) and therefore cannot directly be applied to the unseen trials in the test data. We therefore apply a post-hoc procedure (right hand side of diagram) to estimate a suitable set of activation pattern timings for the test set in an unbiased way. This applies training a linear regression model (top right) to estimate a relationship between the training data and its corresponding activation pattern timings. Note that we train a distinct set of regression weights for each timepoint within the trial. We then apply these regression weights to the test data to obtain estimated activation pattern timings (bottom right). These estimated activation pattern timings can then be used in combination with the previously learned activation patterns to make predictions via the Bayesian decoding model inversion outlined in the text.*

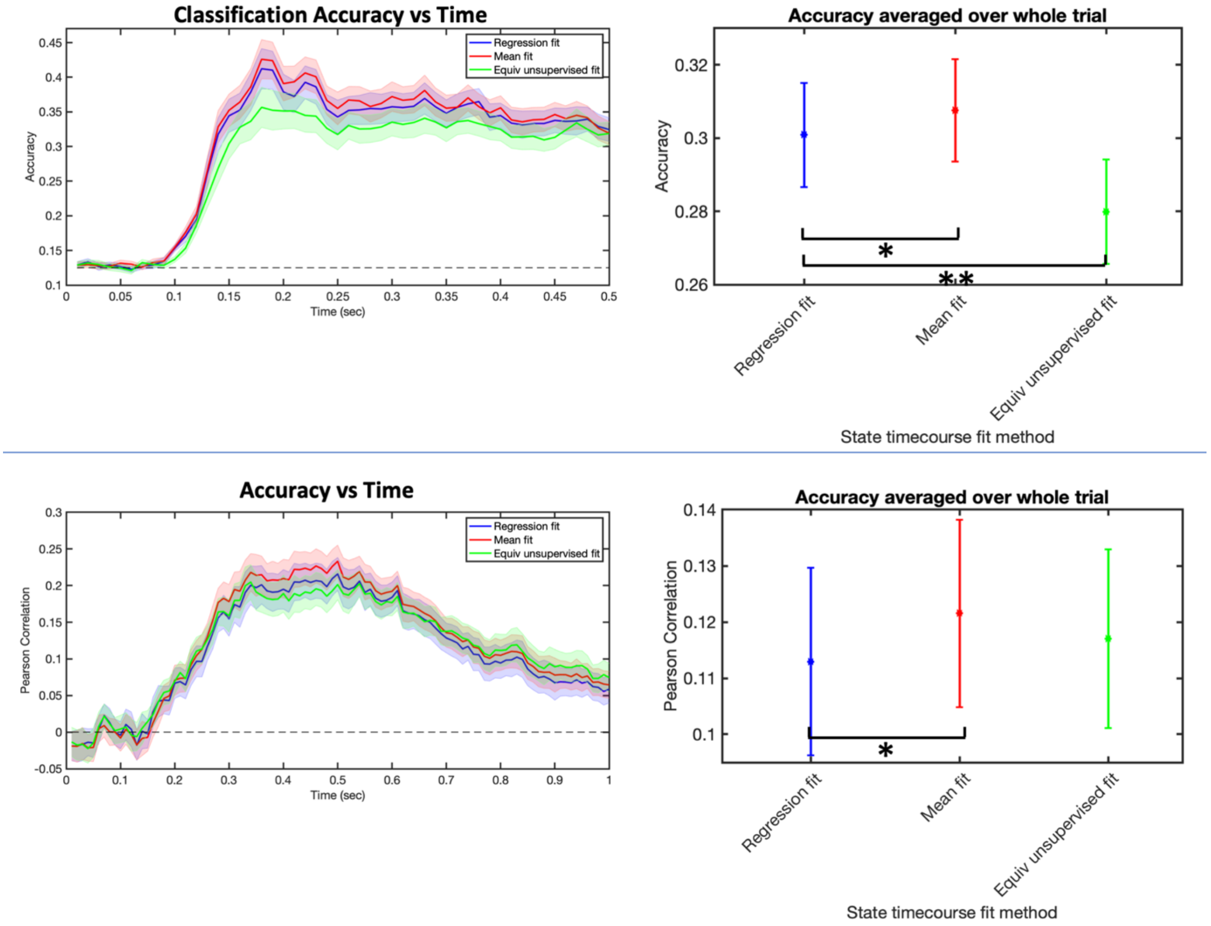

***Figure S4**: **Comparing accuracy of different cross validated state fitting methods**. Top panel: applying different methods to the STRM-Classification paradigm and measuring the classification accuracy (mean over subjects +/- ste), either versus time (left) or averaged over all timepoints (right). Using the mean fit method provided a slight improvement on the regression fit method; using the equivalent unsupervised fit method was significantly worse. A similar result was obtained on the STRM-regression paradigm: we find an improvement when using the mean fit method over the regression fit method; and no significant difference between the regression and equivalent unsupervised fit methods.*

Fitting an equivalent unsupervised model tests whether the same information that determines stimulus activation pattern timings in the STRM model could be reflected more generically with a dynamic model that is common over all stimuli. The relatively poorer performance of the unsupervised HMM model confirms the STRM model is indeed using stimulus specific information in determining state activation timings, and that this timing information is likely reflected in distributed subtle patterns of variation that are not well reflected by unsupervised modelling.

Applying the mean state timecourse effectively ignores any time-varying information in the held out test set, applying a common pattern across all trials. The slightly improved performance achieved by doing so – especially when taken together with the improved performance of optimised sliding window methods

presented in section 3.1.3 and 3.2.3 – suggests either that the methods we have used in cross validation are outputting a poor estimate of state timing information, or that this information is not actually that helpful for classification accuracy. We should note that in our own experimentation we tried quite an exhaustive list of alternative methods for state timing estimation, none of which consistently outperformed the mean fit on the above metrics. Furthermore, as outlined in the main text, the gain in accuracy shown by the HMM model can equivalently be achieved and for some cases overcome by optimised sliding window techniques, that by design have no sensitivity to timing difference across different trials. Thus, as outlined in the text, we have tentatively concluded that knowledge of exact state timing information – which we have shown to reliably correlate with behavioural variables – is not actually particularly informative for improving classification accuracy. There are a number of reasons why this could be the case.

Firstly, the mutual exclusivity assumption imposed by the STRM model is a very strong assumption. It is methodologically quite useful, allowing characterisation of successive stages of processing, but discretising brain activity in this way is potentially counterproductive when assessing classification accuracy over an entire trial. Importantly, when fitting the mean state timecourse, one fits a smoothly averaged mixture of different states at any point in time – transitions between states tend to be much smoother than in the alternative regression fit model – which could achieve more consistent performance if the underlying neural activity trajectories are themselves smoothly varying rather than discrete. Secondly, it is possible simply that the signal to noise ratios are not sufficient to estimate these state timecourses on held out trials. The poor performance of the equivalent unsupervised models suggest the information for these timings is distributed subtle patterns of variation – it is possible that these are simply below some threshold needed to be sufficiently estimated from the data without knowledge of the stimulus.

Finally, one may ask why we have used the regression fit model in the text if the mean fit in fact achieved a better performance. Given all other analyses focus on the time-varying patterns in the data, we wanted to be very clear on the accuracy associated with such time-varying estimates, which is best reflected by the regression fit model. As discussed above, the mean fit imposes the same state timecourse over all trials, and thus does not reflect the influence of time varying dynamics on predictive accuracy.

3. Model Sensitivity to Number of States

The parameter K controls the number of states, which can be considered a critical gauge of the model resolution and its potential to overfit to the data. In assessing predictive accuracy, we have introduced methods to optimise this parameter by cross validation; unfortunately, in our visualisation and state timing correlation group analysis, such methods are not valid as they determine a different optimal number of states for each subject, thereby rendering the underlying interpretation of – for example – the parameters for state 5 inconsistent over subjects. We therefore only use these optimisation methods for the accuracy measurements, and in other methods arbitrarily set $K = 8$. Importantly, we here show evidence that none of these results are dependent on this specific choice of parameter, specifically that the qualitative conclusions drawn from figures 5, 6, 8 and 9 can also be drawn if one selected either $K = 6$ or $K = 10$.

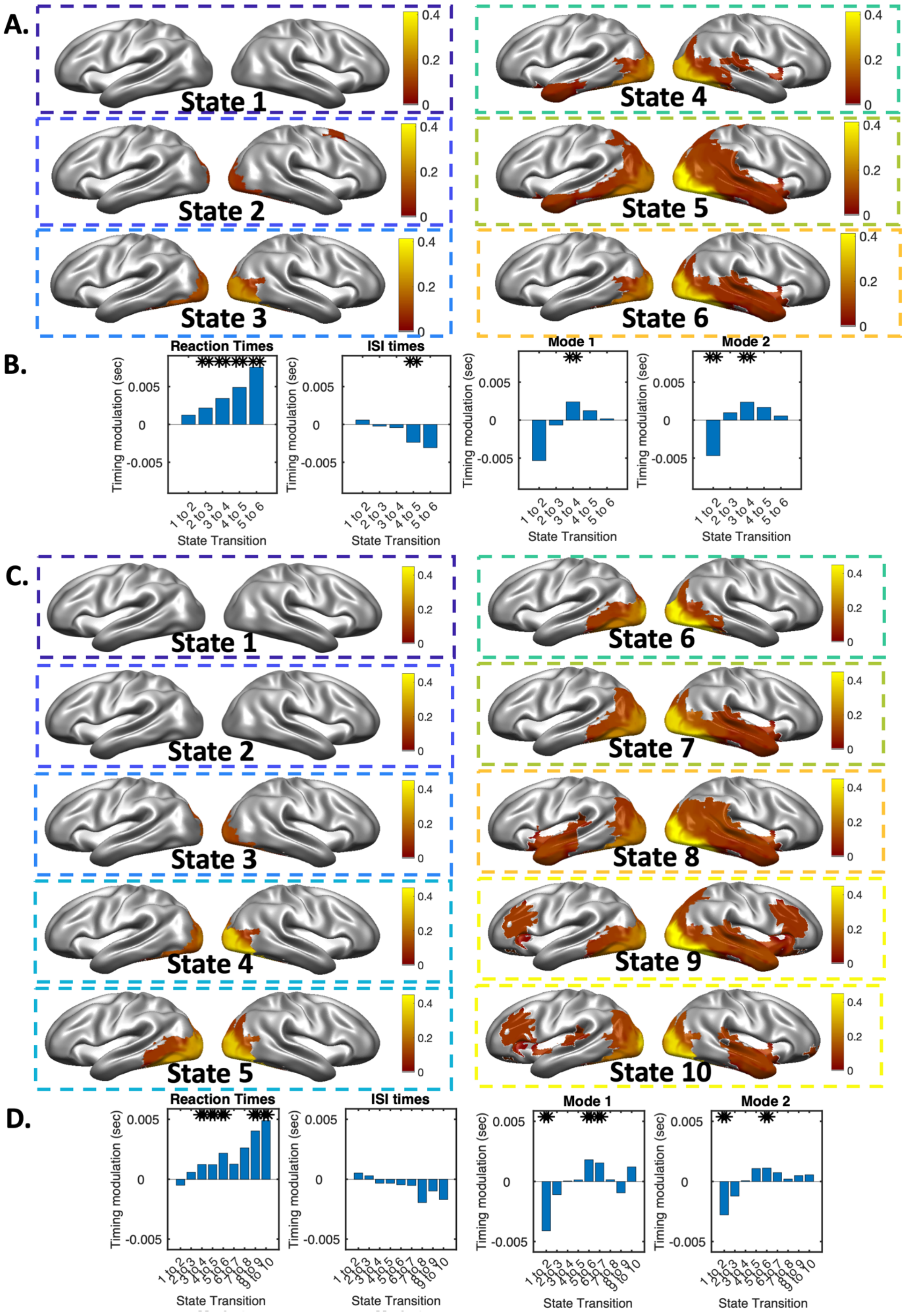

Figure S5: Robustness of STRM-Classification over different parameter values. A. *Replicating the result of figure 5 using K=6 reproduces the same qualitative results, specifically a propagation of information content*

along the visual hierarchy starting with lower visual areas in early states and reaching temporal areas in later states. ***B.*** *Replicating the main result of figure 6 using K=6 again replicates the main result that state timings are modulated by behaviour and physiology; slower reaction times correlate with later propagation through the states, whereas increased pre-stimulus power in the two modes identified in figure 6 are associated with faster transitions into earlier states and slower transitions into later (non-visual) states.* ***C.*** *Replicating the result of figure 5 using* $K = 10$ *again replicates the main finding that information propagates from lower visual areas to a later combination of visual and temporal areas.* ***D.*** *Replicating the result of figure 6 using* $K = 10$ *reproduces the same qualitative conclusions regarding the effects of reaction times and prestimulus power on state transition timings.*

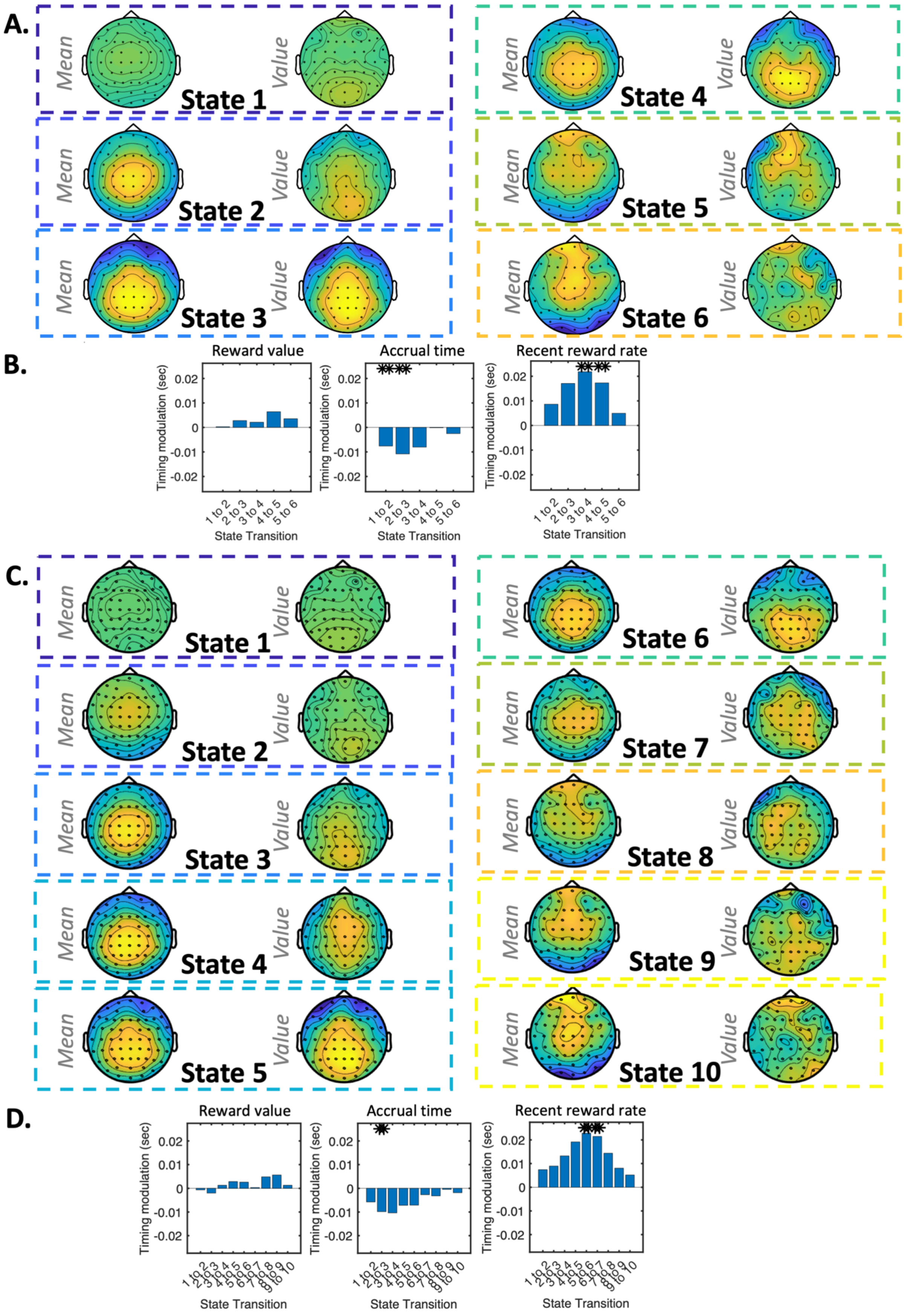

Figure S6: Robustness of STRM-Regression over different parameter values. A. *Replicating the result of figure 8 using K=6 reproduces the same qualitative results, specifically a mean pattern of activation starting over*

parietal areas and propagating slightly towards more frontal areas, with a similar expressed by the evoked value signal albeit slightly delayed relative to the mean evoked response. ***B.*** *Replicating the main result of figure 9 using K=6 again replicates the main result that state timings are modulated by key cognitive variables; the reward values themselves do not significantly affect state timing, however the accrual time modulates the timing of early states whilst the recent reward rate modulates the timing of later states.* ***C.*** *Replicating the result of figure 8 using* $K = 10$ *again replicates the main dynamics.* ***D.*** *Replicating the result of figure 9 using* $K = 10$ *reproduces the same qualitative conclusions regarding the effects of different cognitive variables on state transition times.*

4. Comparison with Inverted Decoding Model predictions

A seminal work by Haufe et. al. identified a popular way to map from any linear decoding model to an equivalent encoding model. Our work effectively argues for the reverse – i.e. fitting an encoding model that can then be mapped to an equivalent decoding model. We here provide some evidence that the direct fitting of an encoding model may in general be more reliable than the post-hoc procedure proposed by Haufe et al. (2014), building upon the established results of (Ng & Jordan, 2002). We make this argument by simulating data, firstly from the generative model of equation 2 (i.e. the exact model of the STRM classifier and of LDA classifiers); and then from a model that violates the assumptions of the STRM classifier and LDA classifiers. In both cases we compare the performance of generative classifiers (in this case LDA) and discriminative classifiers (in this linear SVM) on two metrics; the classification accuracy (i.e. decode model accuracy) and the correlation between the forward model parameters and the known ground truth (i.e. encoding model accuracy).

4.1. Results

Figure S5B replicates the findings of Ng & Jordan (2002), showing that the classification accuracy achieved by an encoding model (i.e. a "generative classifier" in the machine learning terminology, in this case equivalent to an LDA classifier) has the same performance limit as a discriminative classifier (in this case a linear support vector machine classifier), however it approaches this limit faster as a function of the number of training samples. Figure S5C supports our claim that this slight performance deterioration is similarly reflected in the inferred encoding model parameters, for which the forward model parameters obtained using the method of Haufe et al. (2014) shows a slightly higher error than those obtained by fitting a forward model directly.

Importantly this is simulating data from the exact generative model of our classifier, representing quite a narrow and specific case where our arguments might be expected to hold. We therefore ran a second simulation using a model that did not match the generative model of our classifier – specifically by letting one of the classes have a bimodal distribution, as visualised in figure S5D, such that our encoding model assumptions of Gaussian residuals were a poor fit for the data. In this case, again replicating the work of Ng & Jordan (2002), we find that discriminative classifiers converge to a better classification accuracy performance, as they are less constrained by poor modelling assumptions. This does not however translate to more accurate forward model parameters; when the method of Haufe et al. (2014) is applied to these classifiers, the inferred forward model parameters show a greater error relative to those obtained by fitting a forward model directly. This difference in performance, while modest, remains stable over the number of training samples.

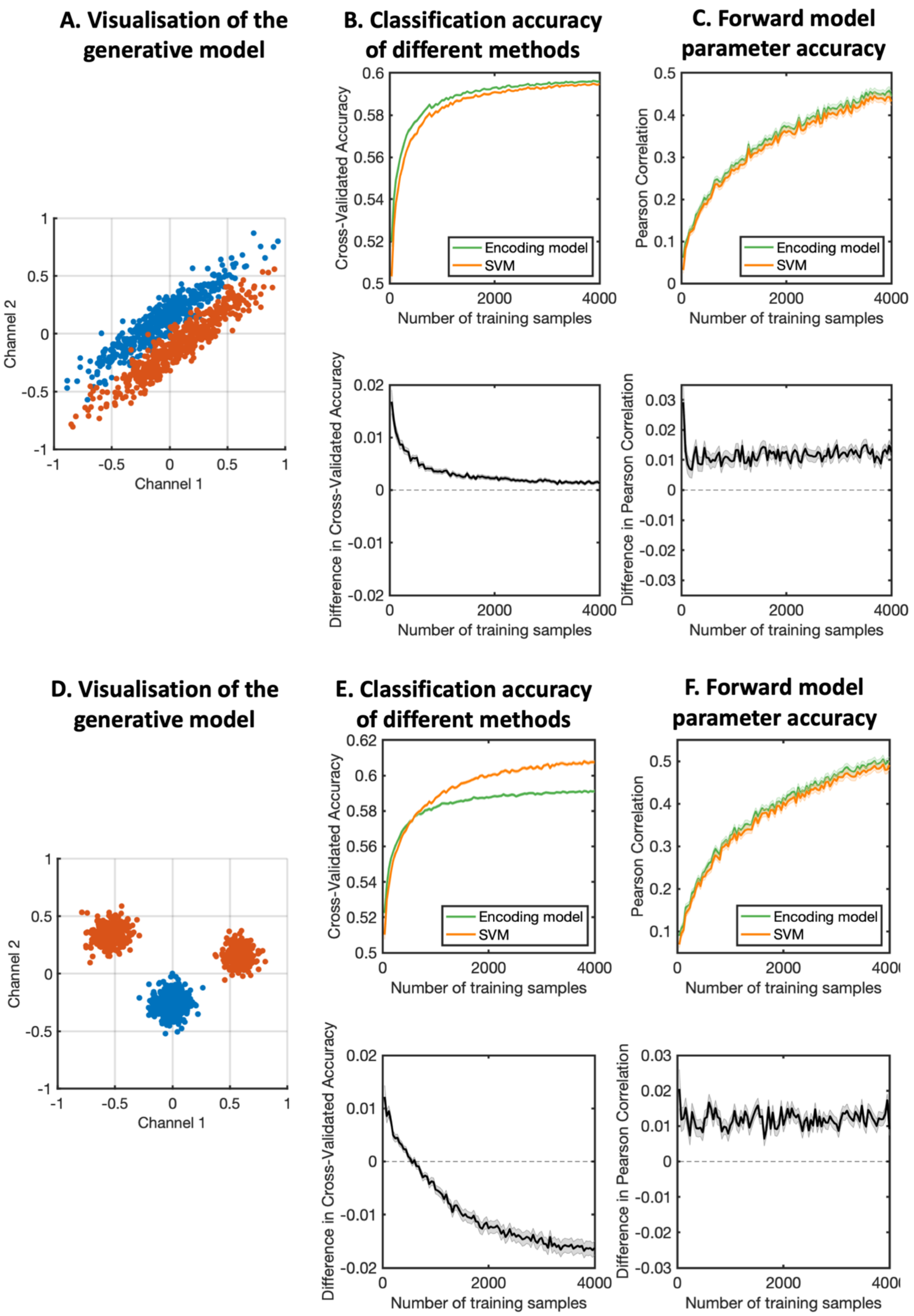

Figure S7: Comparing forward model parameters computed directly or using post-hoc methods applied to linear classifiers. *A. We simulated data from the generative model of an LDA classifier; here shown in two dimensions with high SNR for ease of visualisation but in practice simulated at higher dimensionality with poorer SNR. B. Replicating the established result of* (Ng & Jordan, 2002)*, we find that both classifiers converge*

to the same asymptotic performance level, but that the generative classifiers approach it faster as a function of their accurate (in this case) modelling assumptions. Lower panel plots accuracy of the encoding model classifier minus the accuracy of the decoding model classifier (SVM). C. We can similarly compare the accuracy of the forward model, with the equivalent model parameters for the SVM obtained using the method of Haufe et al. (2014). *This shows a similar profile, with encoding model parameters having slightly higher correlation with the ground truth than those obtained from inverting the decoding model. Lower panel shows the difference in accuracy metrics. D. To test whether this is merely limited to cases that perfectly match the generative modelling assumptions, we simulated data where these assumptions were violated by letting one class have a multi-modal distribution (see simulation details below). Data is plotted here in two dimensions with high SNR for visualisation purposes, in practice the data was simulated in a higher dimensional space with lower SNR. E. Again replicating the findings of* (Ng & Jordan, 2002)*, we see that LDA classifiers reach a lower performance limit than discriminative classifiers, thereby performing worse for all but very low numbers of training samples. This is a result of their modelling assumptions no longer matching the data. F. Despite this, they continue to show better performance if this is measured by their forward encoding model parameters, which show a greater correlation with the ground truth than those recovered by inverting the SVM model parameters using the methods proposed by* Haufe et al. (2014).

4.2. Simulation details

In figure S5A-C, we have simulated data from the generative model of equation 1 (i.e. exactly the generative model of LDA classifiers). Specifically, we simulate data across $P = 20$ sensors in a paradigm with $Q = 1$ regressors, where the single regressor denotes the class and takes values ± 1. These parameters are fixed; on each simulation we randomly draw the condition specific activation pattern W from the standard normal distribution and the residual covariance Σ from a Wishart distribution with scale matrix $\frac{1}{20} I_{20}$ (where I_{20} is the identity matrix over the 20 channels) and shape parameter 20. We then multiply this covariance by a scalar factor to ensure that Fisher's linear discriminant (which uniquely determines the theoretical optimal classification accuracy for this model) is constant over different simulations (fixed to correspond to a theoretical maximum classification accuracy of 0.6). We then split the data into two equally sized folds, use the first to train both an LDA generative classifier and a linear SVM classifier; then test these classifiers on the held-out test fold of the data. At the same time, we take the encoding model parameters used to train the LDA classifier, and similarly the equivalent forward model parameters obtained using Haufe's method from the SVM parameters, and compute the Pearson correlation between these and the ground truth for that simulation. This process was repeated 1000 times whilst the number of training samples was varied from 40 to 4000.

In figure 5D-F we then simulate a less restrictive example where the data no longer perfectly fits the generative model of equation 1. We simulate one of the classes as instead deriving from a multimodal distribution; specifically, we randomly draw an offset from the standard normal distribution, then half of the

samples in this class have that offset added to their data and the other half have that offset subtracted from their data. We then apply the steps as previously outlined to compute the cross validated accuracy and forward model parameter ground truth correlation.

Supplementary Information – References

Beal, M. J. (2003). Variational algorithms for approximate Bayesian inference. In *PhD Thesis* (Issue May).

Haufe, S., Meinecke, F., Görgen, K., Dähne, S., Haynes, J. D., Blankertz, B., & Bießmann, F. (2014). On the interpretation of weight vectors of linear models in multivariate neuroimaging. *NeuroImage*, *87*, 96–110. https://doi.org/10.1016/j.neuroimage.2013.10.067

Higgins, C. (2019). Uncovering temporal structure in neural data with statistical machine learning models. In *Doctoral Thesis*. University of Oxford.

Ng, A. Y., & Jordan, M. I. (2002). On discriminative vs. generative classifiers: A comparison of logistic regression and naive bayes. *Advances in Neural Information Processing Systems*, *14*(841). https://doi.org/10.1007/s11063-008-9088-7

Vidaurre, D., Quinn, A. J., Baker, A. P., Dupret, D., Tejero-Cantero, A., & Woolrich, M. W. (2016). Spectrally resolved fast transient brain states in electrophysiological data. *NeuroImage*, *126*, 81–95. https://doi.org/10.1016/j.neuroimage.2015.11.047
